# Molecular Mechanisms of Resistance to Ceftazidime/Avibactam in Clinical Isolates of *Enterobacterales* and Pseudomonas aeruginosa in Latin American Hospitals

**DOI:** 10.1128/msphere.00651-22

**Published:** 2023-03-06

**Authors:** María Fernanda Mojica, Elsa De La Cadena, Juan Carlos García-Betancur, Jessica Porras, Isabella Novoa-Caicedo, Laura Páez-Zamora, Christian Pallares, Tobias Manuel Appel, Marcela A. Radice, Paulo Castañeda-Méndez, Ana C. Gales, José M. Munita, María Virginia Villegas

**Affiliations:** a Grupo de Investigación en Resistencia Antimicrobiana y Epidemiología Hospitalaria, Universidad El Bosque, Bogotá, Colombia; b Department of Molecular Biology and Microbiology, School of Medicine, Case Western Reserve University, Cleveland, Ohio, USA; c Veterans Affairs Medical Center for Antimicrobial Resistance and Epidemiology (Case VA CARES), Case Western Reserve University-Cleveland, Cleveland, Ohio, USA; d Research Service, VA Northeast Ohio Healthcare System, Cleveland, Ohio, USA; e Departamento de Microbiología, Inmunología, Biotecnología y Genética, Cátedra de Microbiología, Universidad de Buenos Aires—CONICET, Buenos Aires, Argentina; f Department of Infectious Diseases, Hospital Médica Sur, Ciudad de México, México; g Department of Infectious Diseases, Hospital San Angel Inn Universidad, Ciudad de México, México; h Department of Internal Medicine, Division of Infectious Diseases, Universidade Federal de São Paulo, São Paulo, Brazil; i Genomics and Resistant Microbes (GeRM) Group, Millennium Initiative for Collaborative Research on Bacterial Resistance (MICROB-R), Santiago, Chile; Antimicrobial Development Specialists, LLC

**Keywords:** ceftazidime/avibactam, *Pseudomonas aeruginosa*, *Enterobacterales*, antimicrobial resistance, Latin America

## Abstract

Ceftazidime-avibactam (CZA) is the combination of a third-generation cephalosporin and a new non-β-lactam β-lactamase inhibitor capable of inactivating class A, C, and some D β-lactamases. From a collection of 2,727 clinical isolates of *Enterobacterales* (*n =* 2,235) and P. aeruginosa (*n =* 492) that were collected between 2016 and 2017 from five Latin American countries, we investigated the molecular resistance mechanisms to CZA of 127 (18/2,235 [0.8%] *Enterobacterales* and 109/492 [22.1%] P. aeruginosa). First, by qPCR for the presence of genes encoding KPC, NDM, VIM, IMP, OXA-48-like, and SPM-1 carbapenemases, and second, by whole-genome sequencing (WGS). From the CZA-resistant isolates, MBL-encoding genes were detected in all 18 *Enterobacterales* and 42/109 P. aeruginosa isolates, explaining their resistant phenotype. Resistant isolates that yielded a negative qPCR result for any of the MBL encoding genes were subjected to WGS. The WGS analysis of the 67 remaining P. aeruginosa isolates showed mutations in genes previously associated with reduced susceptibility to CZA, such as those involved in the MexAB-OprM efflux pump and AmpC (PDC) hyperproduction, PoxB (*bla*_OXA-50-like_), FtsI (PBP3), DacB (PBP4), and OprD. The results presented here offer a snapshot of the molecular epidemiological landscape for CZA resistance before the introduction of this antibiotic into the Latin American market. Therefore, these results serve as a valuable comparison tool to trace the evolution of the resistance to CZA in this carbapenemase-endemic geographical region.

**IMPORTANCE** In this manuscript, we determine the molecular mechanisms of ceftazidime-avibactam resistance in *Enterobacterales* and P. aeruginosa isolates from five Latin American countries. Our results reveal a low rate of resistance to ceftazidime-avibactam among *Enterobacterales*; in contrast, resistance in P. aeruginosa has proven to be more complex, as it might involve multiple known and possibly unknown resistance mechanisms.

## INTRODUCTION

*Enterobacterales* and the nonfermenting bacilli P. aeruginosa are among the most common pathogenic microorganisms that have acquired resistance to several antibiotic classes (1). The dissemination of β-lactam resistance determinants among these bacteria has radically decreased the effectiveness of last-generation β-lactams, including cephalosporins, carbapenems, and therapeutic combinations with β-lactamase inhibitors. The accumulation of resistance mechanisms to β-lactams and some other antibiotic families significantly hinders the treatment of infections, and obliges the use of less effective and more toxic antibiotics such as colistin and aminoglycosides (1, 2).

The most effective resistance mechanism to carbapenems in Gram-negative pathogens is the production of carbapenemases. In *Enterobacterales*, many class A β-lactamase-encoding genes can yield a carbapenem resistant phenotype. However, *bla*_KPC-2_ and *bla*_KPC-3_ are the most common transmissible genes circulating worldwide and, notably, are endemic to some geographical areas such Latin America (3, 4). In P. aeruginosa, resistance to carbapenems can be achieved either by the hyperproduction of the chromosomal cephalosporinase AmpC or by the production of acquired carbapenemases, particularly of class B metallo-β-lactamases (MBL) such as VIM-2. In addition, nonenzymatic mechanisms such as the modification or inactivation of the porin OprD, or the upregulation of different chromosomally encoded efflux pumps, are also common (5–7).

In the last few years, novel β-lactams/β-lactamase inhibitor combinations are available for the treatment of infections caused by carbapenem resistant *Enterobacterales* and carbapenem resistant P. aeruginosa (8). Among them, ceftazidime-avibactam (CZA) is the combination of an extended-spectrum cephalosporin and a diazabicyclooctane (DBO)-based, non-β-lactam β-lactamase inhibitor. Avibactam is capable of inhibiting the majority of KPC enzymes, including the most wide-spread types, KPC-2 and KPC-3, in addition to other class A β-lactamases; class C cephalosporinases; and to a various degree class D β-lactamases, like some members of the OXA-48 family. However, avibactam cannot inhibit any class B MBL (9).

Resistance to CZA has been extensively reported (1, 2, 6, 10–13). Most cases of CZA resistance in *Enterobacterales*, especially in Klebsiella pneumoniae, have been associated with amino acid substitutions in KPC-2 and KPC-3, particularly the D179Y substitution in the Ω-loop (14–16). Recently, K. pneumoniae isolates resistant to CZA due to the production of KPC-31 (D179Y) and KPC-115 (L168P, ΔAsp169, ΔSer170) were reported causing an outbreak during the COVID-19 pandemic in Argentina (17). CZA resistance due to mutations in *bla*_CTX-M-14_ and *bla*_CTX-M-15_ has also been described in *Enterobacterales* (18–20). For P. aeruginosa resistance to CZA is commonly associated with the presence of amino acid substitution in the Ω-loop of the pseudomonal-derived cephalosporinase (PDC), overexpression of PDC and genetic loss of *oprD*, overexpression of *bla*_OXA-50-like_ β-lactamases, and duplication in the *bla*_OXA-2_, which codes for OXA-539, among others (8). Furthermore, production of ESBLs such as PER-1, which particularly shows weaker kinetic inhibition constants for avibactam, and the presence of tandem *bla*_GES-19_ and *bla*_GES-26_, have been also associated with resistance to CZA (8, 21, 22).

Previously, our group determined the rates of susceptibility to CZA and other relevant antibiotics of clinical *Enterobacterales* isolates collected prior to the introduction of this antibiotic into the clinical practice in Latin America. The resistance rate found in that study was 4.2% (23). Herein, we reassess the phenotypic resistance to CZA of the 94 CZA-resistant *Enterobacterales* strains identified in that previous study; describe the phenotypic resistance rates to CZA of 492 P. aeruginosa clinical isolates collected between 2016 and 2017; and explore the molecular mechanisms leading to CZA resistance in these clinical isolates using whole-genome sequencing (WGS).

## RESULTS

### Molecular characterization of CZA-resistant *Enterobacterales*.

To compare the data previously published for the *Enterobacterales* collection with the new data on the P. aeruginosa isolates from this study, we checked the susceptibility to CZA of 94 isolates previously identified as CZA-resistant. However, after analyzing together the MIC data with Etest, only 18 isolates were confirmed to be truly CZA-resistant. Therefore, the updated CZA resistance rate of this collection of *Enterobacterales* is 0.8% (18/2,235). Of interest, all 18 CZA-resistant isolates were collected in Colombia, at different times, from nine medical centers located in nine cities. For *Enterobacterales*, we expanded the battery of tests performed before, adding the RAPIDEC Carba-NP assay to detect carbapenemase activity, and qPCR to confirm the presence of at least one MBL-encoding gene in these isolates (Table 1). Furthermore, three isolates of K. pneumoniae and one Enterobacter cloacae complex co-harboring *bla*_NDM_ and *bla*_KPC_ were detected. Since resistance to CZA is explained by the presence of at least one MBL-encoding gene in CZA-resistant *Enterobacterales*, WGS was not performed on any of these isolates.

**TABLE 1:** Breakdown of ceftazidime-avibactam resistance rates among *Enterobacterales* and their associated resistance genes

Bacterial species	No. of isolates tested	Resistant isolates	Positive isolates for Carba-NP	Positive isolates qPCR (%)			
		n (%)		*bla*_KPC_	*bla*_NDM_	*bla*_KPC_ + *bla*_NDM_	*bla*_VIM_
E. coli	1,397	1 (0.07)	1	0 (0)	1 (0.07)	0 (0)	0 (0)
K. pneumoniae	607	13 (2.1)	13	0 (0)	10 (1.6)	3 (0.5)	0 (0)
E. cloacae complex	112	3 (2.7)	3	0 (0)	1 (0.9)	1 (0.9)	1 (0.04)
S. marcescens	90	1 (1.1)	1	0 (0)	1 (1.1)	0 (0)	0 (0)
K. aerogenes	29	0	0	0 (0)	0 (0)	0 (0)	0 (0)
Total	2,235	18 (0.8)	18	0	13 (0.6)	4 (0.2)	1 (0.04)

### Antibiotic susceptibility and molecular characterization of CZA-resistant P. aeruginosa.

The distribution of the 492 isolates of P. aeruginosa per country is shown in Table 2. Overall, 22.1% (109/492, MIC_50_ 4/4 mg/L, MIC_90_ 64/4 mg/L) of the isolates were resistant to CZA. In addition, complete MIC data are presented in Table S1.

**TABLE 2:** Percentage of resistance of P. aeruginosa to ceftazidime-avibactam and comparator agents by country^*a*^

Country	No. of isolates	CZA		CAZ		FEP		PTZ		IMI		MER	
		n	(%)	n	(%)	n	(%)	n	(%)	n	(%)	n	(%)
Colombia	239	59	(25)	103	(43)	98	(41)	124	(52)	164	(69)	122	(51)
Chile	61	7	(11)	26	(43)	22	(36)	25	(41)	35	(57)	22	(36)
Argentina	29	10	(34.4)	19	(66)	15	(52)	20	(69)	20	(69)	17	(59)
Mexico	124	28	(23)	61	(49)	63	(51)	61	(49)	93	(75)	68	(55)
Brazil	39	5	(13)	13	(33)	22	(56)	20	(51)	34	(87)	28	(72)
Total	492	109	(22.1)	222	(45)	220	(45)	250	(51)	346	(70)	257	(52)

a CZA, ceftazidime avibactam; CAZ, ceftazidime; FEP, cefepime; PTZ, piperacillin tazobactam; IMI, imipenem; MER, meropenem.

10.1128/msphere.00651-22.1 TABLE S1 Minimum inhibitory concentration (MIC) to CZA and additional antimicrobials of P. aeruginosa isolates used in this study. ARG, Argentina; BRA, Brazil; CHL, Chile; COL, Colombia; MEX, Mexico. CZA, ceftazidime avibactam; C/T, ceftolozane tazobactam; CAZ, ceftazidime; COL, colistin; FEP, cefepime; PTZ, piperacillin tazobactam; IMI, imipenem; MER, meropenem; DOR, doripenem; FOS, fosfomycin. Download Table S1, XLSX file, 0.04 MB. Copyright © 2023 Mojica et al. 2023 Mojica et al. https://creativecommons.org/licenses/by/4.0/ This content is distributed under the terms of the Creative Commons Attribution 4.0 International license.

All CZA-resistant P. aeruginosa isolates were then subjected to RAPIDEC Carba-NP test and qPCR. These tests found 42 isolates (38.5%) with MBL (three for *bla*_IMP_; 31 for *bla*_VIM_; one for *bla*_SPM-1_; and seven carrying a combination of *bla*_KPC_ and *bla*_VIM_), and eight positives for *bla*_KPC_. However, 59 did not carry any carbapenemases (Table 3). Notably, the only isolate harboring *bla*_SPM-1_ yielded a negative result in the RAPIDEC Carba-NP assay.

**TABLE 3:** Breakdown of ceftazidime-avibactam resistance rates among P. aeruginosa and their associated resistance genes

Country	No. of isolates tested	Resistant isolates	Positive isolates for Carba-NP	Positive isolates qPCR (%)				
		n (%)		*bla*_KPC_	*bla*_VIM_	*bla*_KPC_ + *bla*_VIM_	*bla*_IMP_	*bla*_SPM-1_
Colombia	239	59 (24.7)	39	8 (3.3)	24 (10)	7 (2.9)	0 (0)	0 (0)
Chile	61	7 (11.5)	3	0 (0)	3 (4.9)	0 (0)	0 (0)	0 (0)
Argentina	29	10 (34.5)	2	0 (0)	0 (0)	0 (0)	2 (6.9)	0 (0)
Mexico	124	28 (22.6)	5	0 (0)	4 (3.2)	0 (0)	1 (0.8)	0 (0)
Brazil	39	5 (12.8)	0 (0)	0 (0)	0 (0)	0 (0)	0 (0)	1 (2.6)
Total	492	109 (22.1)	49	8 (1.62)	31 (6.3)	7 (1.4)	3 (0.6)	1 (0.2)

### WGS analysis of P. aeruginosa isolates resistant to CZA and associated resistance genes.

A total of 67 P. aeruginosa genomes were sequenced. This number corresponds to the 59 isolates that yielded negative results for the multiplex qPCR and eight additional isolates that tested positive for the presence of *bla*_KPC_. Due to unexpected low sequence coverage (<30×), we excluded six samples from the subsequent analysis (five isolates negative for any carbapenemase gene and one positive for *bla*_KPC_). The remaining 61 samples showed quality values over 90%. We obtained between 110 to 137 contigs per isolate sequenced, with a length of the assemblies between 6.3 to 7.2 Kb and, a GC content ranging from 65.8% to 66.5%. Sequencing quality data are presented in Table S2.

10.1128/msphere.00651-22.2 TABLE S2 Illumina quality data of the genome assemblies of the P. aeruginosa isolates sequenced in this study. ARG, Argentina; BRA, Brazil; CHL, Chile; COL, Colombia; MEX, Mexico. Download Table S2, XLSX file, 0.04 MB. Copyright © 2023 Mojica et al. 2023 Mojica et al. https://creativecommons.org/licenses/by/4.0/ This content is distributed under the terms of the Creative Commons Attribution 4.0 International license.

**FIG 1:**
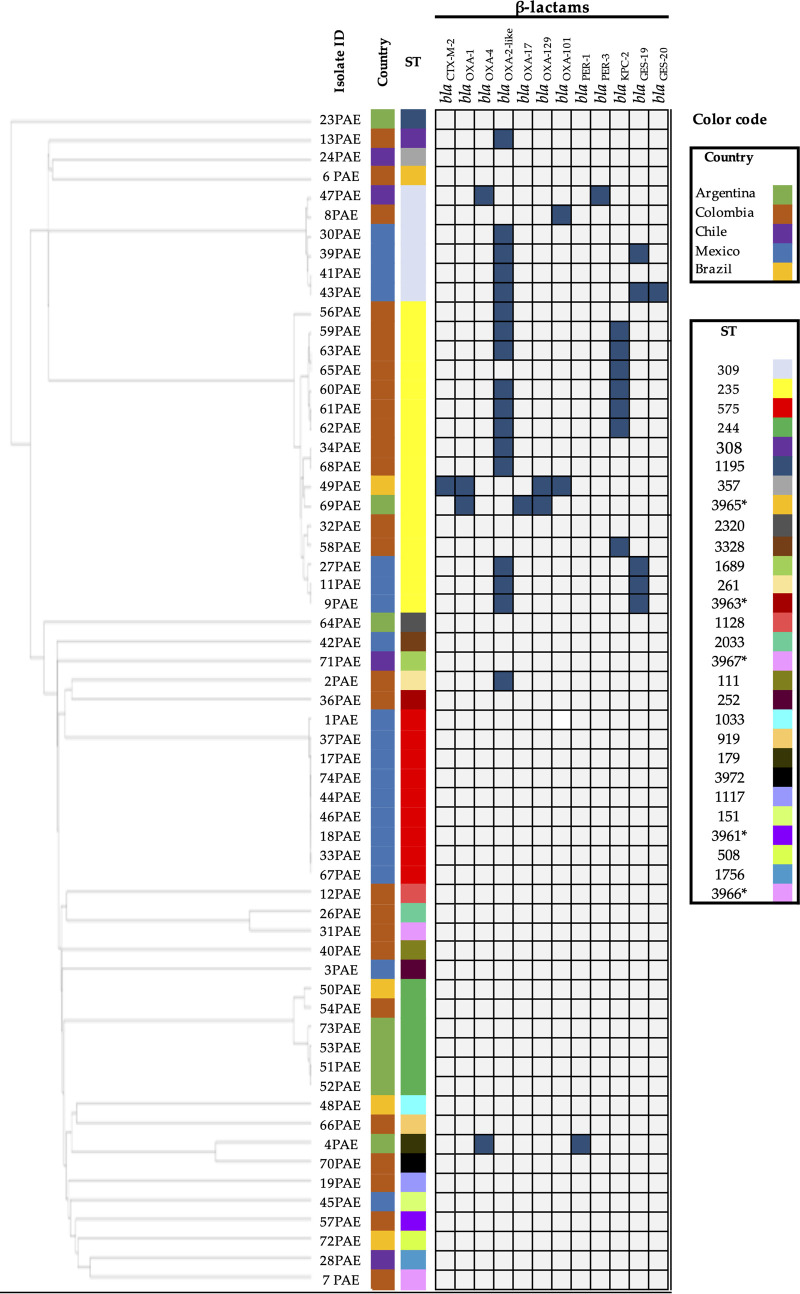
Genetic resistance determinants of CZA-resistant P. aeruginosa isolates. Blue squares represent the presence of the respective antibiotic resistance gene, and blank represents its absence. The phylogenetic tree was obtained by canonical wgMLST using the web server cano-wgMLST_BacCompare (25) and drawn using the iTOL tool (50). ST, sequence type. New ST are indicated by *.

WGS analysis revealed 23 known sequence types (STs), and five new STs, as shown in Fig. 1. Relevant STs found included ST111 (*n *= 1) and ST308 (*n *= 1) from Colombia; ST357 (*n *= 1) from Chile; and ST309 (*n *= 6) were found in four isolates from Mexico, one from Colombia, and one from Chile. Clonal dissemination was observed among some isolates: ST575 (*n *= 9) was only reported in Mexico; ST235 (*n = *16) in Colombia, Mexico, Brazil, and Argentina; and ST244 mainly in Argentina.

Confirming their species identity, sequence analysis of the P. aeruginosa genomes showed that all of them carried *bla*_AmpC_ and *bla*_OXA-50-like_. From the 61 genomes analyzed, 17 (27.9%) harbored *bla*_OXA-2_: 11 isolates belonging to the ST235 from Mexico and Colombia, four isolates with ST309 from Mexico, and two belonging to the ST308 and ST261 isolated from Colombia (Fig. 1). However, none of the evaluated isolates harbored mutations in *bla*_OXA-2_, including duplication in the *bla*_OXA-2_, which encodes for OXA-539.

Three isolates from Mexico belonging to the ST235 and one belonging to the ST30 harbored *bla*_GES-19_. Interestingly, one isolate belonging to the ST309 from Mexico harbored *bla*_GES-19_ and *bla*_GES-20_ in tandem. Also, one isolate from Argentina and one from Chile, were found to harbor *bla*_PER-1_ and *bla*_PER-3_, respectively. All sequenced isolates harboring *bla*_KPC-2_ (*n *= 7) were isolated in Colombia and belonged to the high-risk clone ST235. To note, none of these isolates showed mutations in *bla*_KPC-2_.

To explore in detail the molecular mechanisms previously associated with resistance to CZA in these P. aeruginosa clinical isolates, we analyzed a variety of genes for any mutation that could lead to overexpression or repression of a particular gene, or to amino acid substitutions that could change the activity of the protein. These genes include β-lactamase encoding genes (e.g., *bla*_PDC_) and their regulator genes (*bla*_AmpD_, *bla*_AmpR_, *bla*_AmpG_); genes encoding the multidrug efflux MexA-B, and its regulators (MexR, NalC and NalD); (*ftsI*, and *dacB* encoding PBP3 and PBP4, respectively); *creD*, which encodes a predicted inner membrane protein part of the conserved two-component regulatory system CreBC (24); and genes involved in pathogenesis like DnaJ, DnaK, and ATP-dependent Clp protease proteins (13, 25–27).

Specifically, predicted substitutions in AmpG, DnaJ, DnaK, and ATP-dependent Clp protease proteins were not found. The proteins that had substitutions in most isolates were PDC, PoxB/OXA-50-like, NalC, and CreD. Most of the proteins had multiple substitutions, except peptidases S41, PBP3/FtsI and NalD, which had only one substitution in some isolates (Table S3). Substitutions in MexAB-OprM regulator proteins, most frequently a G71E change in NalC (77%) and a V126E substitution in MexR (47.5%) were observed. Mutations leading to substitutions in PBP3, PoxB, and the PDC/AmpC system were detected in 9.8%, 95.1%, and 82% of the P. aeruginosa CZA-resistant isolates, respectively. Only six isolates had the substitution N117S in PBP3, all of them belonging to the ST309 from Mexico (four), Colombia (one), and Chile (one) (Table S3).

10.1128/msphere.00651-22.3 TABLE S3 Principal genomic characteristics associated with CZA-resistance found in the P. aeruginosa isolates sequenced. ARG, Argentina; BRA, Brazil; CHL, Chile; COL, Colombia; MEX, Mexico. Download Table S3, XLSX file, 0.07 MB. Copyright © 2023 Mojica et al. 2023 Mojica et al. https://creativecommons.org/licenses/by/4.0/ This content is distributed under the terms of the Creative Commons Attribution 4.0 International license.

Of special interest, clonal spread of the mutations linked to particular STs was observed in our results. In all isolates belonging to the ST235 recovered from Colombia, Argentina, and Mexico, we found identical substitutions in PDC (G1D, A71V, T79A, V179L, and G365A), AmpG (A583T), AmpR (G283E, M288R), and AmpD (G148A). Similarly, in all isolates belonging to the ST244 from Argentina, Brazil, and Colombia, identical substitutions were observed in CreD (Q253E, A394V, F445L, R451K, I469A), AmpD (G148A, D183Y), AmpG (A583T), and PoxB (L6F, R49C), compared to P. aeruginosa PAO1. Likewise, strains belonging to the ST309 from Mexico, Chile, and Colombia had identical substitutions in CreD (D95N, V335I, A394V, F445L, and I469A), AmpG (A583T), AmpR (G283E, M288R), MexA (K16K), MexR (V126E), and PBP3 (N117S). Lastly, isolates belonging to ST575, all isolated in Mexico, had the same substitutions in DacB (A394P), CreD (F445L, R451K, and I469A), AmpG (A583T), PDC (T79A), OprD (D43L, S57E, S59R, E202Q, I210A, E230K, S240T, N262T, A267S, A281G, K296Q, and Q300E), and MexR (Δ1-4aa) (Table S3).

## DISCUSSION

In a previous study, we evaluated the *in vitro* activity of CZA against a set of 2,252 clinical isolates of *Enterobacterales* in Latin America, finding that 4.2% were resistant (23). However, combined phenotypic tests performed in this study confirmed the CZA-resistant phenotype of only 18/94 isolates. Therefore, the updated resistance rate to CZA of this group of *Enterobacterales* is 0.8%. Additionally, we analyzed the susceptibility to CZA of a set of 492 clinical isolates of P. aeruginosa collected during the same time period (2016 to 2017) in the same five Latin American countries. Finally, we determined the molecular mechanisms leading to CZA resistance in these isolates by WGS.

Several molecular mechanisms leading to decrease susceptibility to CZA have been described in P. aeruginosa. Among them, specific amino acid substitutions in some β-lactamases, including KPC and SHV have been associated with resistance to CZA (11). In particular, the D179Y substitution in the Ω-loop of KPC-3, and in other KPC variants, confer resistance to CZA. Of note, this mechanism was reported in a P. aeruginosa isolate from Chile before this antibiotic was clinically available in this country (28). Interestingly, all sequenced P. aeruginosa isolates that carried *bla*_KPC-2_ retrieved in Colombia belonged to ST235. This ST has been associated with the disseminations of *bla*_KPC-2_ in Colombia (2). As we did not evidence any mutations in *bla*_KPC-2_, CZA-resistance is most probably caused by other mechanisms. All of these seven strains (58PAE to 63PAE and 65PAE in Table S3) have multiple mutations in several genes, including in *ampR* leading to the substitutions G283E, M288R in AmpR, and mutated *ampG*, producing the variant A583T. The association of these mutations with CZA resistance is yet to be determined. Moreover, six out of seven isolates showed mutations in *nalD* (coding for the MexAB-oprM regulator), which could lead to decreased susceptibility to CZA as previously reported (29, 30) (Table S3).

Regarding the molecular epidemiology, WGS analysis revealed that some of the CZA-resistant P. aeruginosa isolates belonged to ST235 (*n* = 16), ST244 (*n* = 6), and ST111 (*n* = 1). These STs have been considered as high-risk clones (31, 32). Furthermore, ST235 and ST111 are multidrug resistant (MDR) clones disseminated worldwide and linked to the expression of VIM-2 (2). Sixteen of the sequenced isolates belonging to ST235 did not harbor any *bla*_VIM_ gene but all harbored *bla*_KPC_. A surveillance study of P. aeruginosa performed in Colombia found that ST111 is a common host of *bla*_VIM-2_, whereas ST235 is associated with *bla*_KPC-2_, as aforementioned (33). Additionally, an isolate that carried *bla*_SPM-1_ belonged to ST277, which is a ST commonly associated with the dissemination of *bla*_SPM-1_ in Brazil (12).

Extended-spectrum β-lactamases (ESBL) such as PER and GES have also been associated with resistance to CZA via biochemical mechanism conferring a weaker inhibitory potency of avibactam to these enzymes (34). This kinetic feature, possibly combined with the lower permeability of P. aeruginosa, can effectively decreased the susceptibility to CZA (9, 13, 34, 35). In our study, one P. aeruginosa isolate from Argentina (ST179) and one isolate from Chile (ST309) harbored *bla*_PER-1_ and *bla*_PER-3_, respectively. In addition, five isolates from Mexico carried *bla*_GES-19_, three of them were ST235 and the other two were ST309. In Mexico, a high prevalence of the ESBL GES-19 and the carbapenemase GES-20 have been reported as the most prevalent in P. aeruginosa (36). Moreover, it has been reported that the presence of the ESBL-encoding genes *bla*_GES-19_ and *bla*_GES-26_ in tandem is associated with resistance to all β-lactams, including CZA (21). Importantly, in the present study one of the P. aeruginosa isolates belonging to ST309 showed a similar feature, where *bla*_GES-19_ and *bla*_GES-20_ were found in tandem, which might explain the resistance to CZA. Dissemination of P. aeruginosa isolates harboring either *bla*_PER_ or *bla*_GES_ genes is worrisome, as production of these enzymes compromise the efficacy of the latest anti-pseudomonal drugs, CZA and ceftolozane-tazobactam (14, 37).

A recent study by Fraile-Ribot et al. found that the duplication of the residue D149 in OXA-2 led to resistance to CZA *in vivo* (8). This new variant of OXA-2, called OXA-539 was reported for the first time in a P. aeruginosa isolate resistant to CZA belonging to ST235, from a patient with a susceptible isolate who was previously treated with CZA (8). In our analysis, 17 P. aeruginosa isolates carried OXA-2, 11 of them belonging to ST235 but none of them had the D149 duplication. Worth noting, all P. aeruginosa resistant to CZA and harboring *bla*_OXA-2_ were exclusively recovered from Mexico and Colombia.

Several enzymes of class D, including PoxB (OXA-50-*like*), which is encoded in the chromosome of all P. aeruginosa strains, are not efficiently inhibited by DBOs (38). Compared to the PoxB encoded in the reference strain P. aeruginosa PAO1, multiple substitutions in PoxB were found in our isolates. However, there is no evidence that these mutations can lead to resistance to CZA. On the contrary, Castanheira et al. described substitutions in PoxB in both susceptible and resistant isolates, suggesting that these changes are not directly leading to CZA-resistance (25).

Although CZA shows potent inhibitory activity against PDC (AmpC) of P. aeruginosa, mutations in *bla*_PDC_ conferring resistance to CZA have been reported (39). Here, we found 14 different PDC variants, being PDC-3, PDC-35, and PDC-1 the most frequent (Table S3). However, these variants have not been associated with a particular antimicrobial resistance pattern in previous studies. Moreover, previous investigations have suggested that amino acid substitutions in the PDC enzyme are unlikely to be the main mechanism conferring resistance to CZA, because a correlation between the PDC enzyme variations and the MIC has not been detected (40). However, the recent emergence of P. aeruginosa clinical isolates overexpressing variants of PDC is worrisome and may compromise the efficacy of CZA (40). Indeed, the E247K, G183D, T96I, and ΔG229 to E247 substitutions and deletions appear to perform a 2-fold effect on the catalytic cycle of PDC, allowing to evade avibactam inhibition, while hydrolyzing ceftazidime with enhanced efficiency (40). More biochemical studies are needed to elucidate the relation between the PDC variants identified in this study and CZA-resistance in P. aeruginosa.

As previously mentioned, changes in PBPs can lead to CZA resistance. For instance, FtsI (PBP3) of P. aeruginosa, is the PBP to which many β-lactams, including monobactams and some cephalosporins, have the highest affinity for. FtsI is the primary target of ceftazidime, however, avibactam is also known to covalently bind to the PBPs of P. aeruginosa (1). The FtsI variants R504C and P527S have been strongly associated with reduced susceptibility to different types of β-lactams, including ceftazidime (5). We did not find these mutations in our isolates. However, six sequenced P. aeruginosa isolates showed the same FtsI variant, N117S, which, has not been associated to CZA resistance, and given its location within the protein, an effect on CZA-resistance is unlikely. Interestingly, all the strains harboring the N17S variant of FtsI belonged to the ST309, which has been described in serious infections involving MDR and XDR P. aeruginosa strains. Furthermore, all six isolates were recovered from different geographical locations Mexico, Colombia, and Chile, suggesting that the geographic distribution of ST309 is widespread (21).

A study from Castanheira et al. showed that MexAB-OprM efflux system overexpression was significantly associated with CZA resistance, alone or in combination with alterations or disruptions in other genes (25). Furthermore, it has been shown that disruption of MexR, a negative regulator of MexAB-OprM, leads to high expression of the MexAB-OprM efflux pump slightly raising the MIC of CZA (41). In our analysis, nine isolates belonging to the ST575 from Mexico showed altered versions of MexR. Additionally, 18 isolates (5 from Argentina [ST244 and ST179], 11 from Colombia [ST235 and ST3963], 1 from Chile [ST357], and 1 from Brazil [ST235]) had mutations, framework-shifts, or alterations in the NalD, a repressor of MexAB. Mutations in NalD have been associated with hyperexpression of MexAB and therefore, resistance of all β-lactams (30).

Regarding the *Enterobacterales*, we determined that the presence of at least one MBL-encoding gene in all evaluated isolates could be the underlying molecular mechanism leading to CZA-resistance. The presence of MBL-encoding genes in CZA-resistant *Enterobacterales* has been frequently reported in the United States, countries of the Asian-Pacific region, and Europe (9, 42, 43).

Interestingly, all CZA-resistant *Enterobacterales* were isolated in Colombia, where KPC-enzymes are considered endemic (44). Although specific amino acid substitutions in the Ω-loop of KPC leading to CZA-resistance in *Enterobacterales* have been reported in several countries, we did not find isolates harboring *bla*_KPC_ without an MBL-encoding gene. Conversely, the prevalence of *Enterobacterales* carrying MBL, especially NDM, either alone or in combination with a serine carbapenemase has increased in recent years in this country (45). Exemplary for this observation, we found four isolates from Colombia harboring both *bla*_KPC_ and *bla*_NDM_ (1, 10, 42).

### Conclusions.

By the time of the collection of these isolates, a low rate of resistance to CZA was found among *Enterobacterales* in the Latin American countries that participated in this study. In this analysis, we demonstrated that the most common mechanism of resistance in *Enterobacterales* was the production of MBLs. In contrast, resistance to CZA in P. aeruginosa has proven to be more complex, as it might involve multiple known and possibly unknown resistance mechanisms.

Our study has many limitations. Due to budget restrictions, we could only sequence some of the CZA-resistant isolates and none of the CZA-susceptible ones. This impeded us to have the complete molecular snapshot of all *Enterobacterales* and P. aeruginosa isolates. Consequently, we are only reporting known mechanisms of reduced susceptibility to CZA in these isolates. More studies are needed to investigate emerging mechanisms of resistance to CZA. Nevertheless, as these isolates were collected before the clinical use of CZA in Latin America, the results presented here offer a valuable tool for upcoming comparisons with isolates of *Enterobacterales* and P. aeruginosa recovered after its introduction in this region. These studies will delineate the evolutionary path of the CZA-resistance and how its use in the clinical practice affects the epidemiology of these MDR pathogens. The knowledge of the evolution of resistance to last-resort antibiotics such as CZA in clinical isolates will help to understand the role of selective pressure in different scenarios.

### Ethical approval.

The protocol was approved by the ethics committee of Universidad El Bosque, under act #018-2020. Collection of the microbiological isolates was part of the regular diagnostic process, as established by each of the participating health care institutions.

## MATERIALS AND METHODS

### Susceptibility testing and detection of carbapenemases.

Resistance to CZA was confirmed by MICs determined by broth microdilution method using customized Sensititer plates (Trek Diagnostic Systems, Thermo Fisher Scientific, UK) following the manufacturer’s recommendations and, Etest (bioMérieux, Marcy l’Etoile, France). Results were interpreted according to the current guidelines of the Clinical and Laboratory Standards Institute (CLSI) (46). Presence of carbapenemases in CZA-resistant *Enterobacterales* and P. aeruginosa isolates was initially screened by RAPIDEC Carba-NP Assay (bioMérieux, Marcy-l'Étoile, France) (47), followed by qPCR targeted to the *bla*_KPC_, *bla*_NDM_, *bla*_VIM_, *bla*_IMP_, *bla*_oxa-48-like_, and *bla*_SPM-1_ genes. The reference strains Escherichia coli ATCC 25922, K. pneumoniae ATCC 700603 and P. aeruginosa ATCC 27853 were used as the quality control strain, as per CLSI recommendations (46).

### Whole-genome sequencing.

Genomic DNA was extracted using DNeasy blood and tissue kit (Qiagen, Hilden, Germany) according to the manufacturer’s protocol. Genomic sequencing of 67 clinical isolates *of* P. aeruginosa were performed on the Illumina MiSeq platform (Illumina Inc., San Diego, California, USA) with 250 nt paired end reads to achieve a coverage of about 30× per base, using MiSeq V3 flow cell. *de novo* assembly were performed using CLC Genomics Workbench 8.1.0 software and annotation was done by using RAST server. Multi locus sequence type (MLST) 1.8 server was used to determine the sequence type (ST) of P. aeruginosa isolates (https://cge.food.dtu.dk/services/MLST/) (48). Additionally, antibiotic resistance genes were predicted using online databases (https://cge.food.dtu.dk/services/ResFinderFG/) (49). The genome of P. aeruginosa PAO1 (GenBank ID: NC_002516.2) was used as reference, in order to look for known alterations and disruptions in proteins involved in efflux, regulation of PDC, PBPs, and others associated with CZA resistance. The proteins analyzed were PDC (AmpC), AmpR, AmpG, AmpD, FtsL (PBP-3), PoxB (OXA-50-like), DacB (PBP-4), CredD, MexA, MexB, MexR, OprD, DnaJ, DnaKATP-dependent Clp protease proteins, NalC and NalD (25).

The analyses of the 61 CZA-resistant P. aeruginosa isolates were conducted using cano-wgMLST_BacCompare web-based tool (http://baccompare.imst.nsysu.edu.tw) (50), while the cano-wgMLST tree was built using the highly discriminatory loci among isolates. The dendrogram was visualized with iTOL v6 (http://itol.embl.de) (51).

### Data availability.

The genome sequencing data are publicly available at NCBI GenBank under the BioProject accession number PRJNA729968.
